# Resolution of Ring Tourniquet with a High-speed Dental Drill in a Remote Pacific Island Clinic

**DOI:** 10.7759/cureus.4474

**Published:** 2019-04-16

**Authors:** Rombod Rahimian, Matthew Lippi, Joseph Rusaqoli, Lidia M Perez

**Affiliations:** 1 Emergency Medicine, University of Arizona, Tucson, USA; 2 Emergency Medicine, University of Colorado, Aurora, USA; 3 Miscellaneous, Aitutaki Hospital, Aitutaki, COK; 4 Obstetrics & Gynecology, Aitutaki Hospital, Aitutaki, COK

**Keywords:** ring tourniquet, dental drill, ring, hand, emergency, ischemia, strangulation

## Abstract

Ring tourniquet syndrome is a strangulation injury, usually at the proximal finger or toe, caused by a rigid circular metal object. The resulting ischemia can lead to necrosis, permanent nerve and/or tissue damage, and amputation of the digit. There are numerous non-cutting methods for removing the ring; however, edema, fractures, or arthrosis of the site can occasionally make these techniques difficult or impossible. While ring cutters, manual or electric, are the first choice for resolving ring tourniquet caused by metal jewelry, these tools are not readily available everywhere. Resolution of ring tourniquet with high-speed rotary tools has been previously described as a tertiary method. Here we describe the use of a high-speed dental tool as a primary ring cutting method for the resolution of ring tourniquet in a patient with significant edema in a low-resource setting.

## Introduction

Ring tourniquet syndrome is a strangulation injury caused by a rigid circular object, most often a piece of jewelry or metal nut. The site of the injury is generally the proximal finger or toe, although there are case reports of ring tourniquet syndrome of the penis and testicles [1]. The strangulation generally occurs secondary to edema from infection, trauma, or other inflammatory processes that leads to entrapment of the ring. The constricting ring worsens the edema by reducing venous and lymphatic drainage and ultimately restricts arterial supply [2]. Without treatment in a timely fashion, tendon and nerve damage, ischemia and necrosis can ensue leading to amputation.

There are multiple well-documented ring sparing and ring cutting techniques for resolving ring tourniquet. If ring cutting methods are required, standard ring cutters will often suffice for softer jewelry metals, such as gold or silver. However, for hardened metals, such as stainless steel or tungsten carbide rings, diamond tipped cutting instruments or shattering the ring with a hammer may be necessary [3].

In this case, a 41-year-old Maori female presented to a rural island health outpost in the Cook Islands with pain and swelling of the left ring finger, which she first noticed three days ago following a ten-hour plane flight. The patient denied any recent trauma or sick symptoms. On examination, the left ring finger demonstrated marked fusiform swelling, with erosion of the skin due to a wedding ring tourniquet. The outpost serves as the only health facility on the island of Aitutaki, providing primary medical and dental services. The clinic did not have manual or electric ring cutters available. Here, we present the successful resolution of a ring tourniquet using a high-speed dental drill in a rural health clinic.

## Case presentation

**Figure 1:**
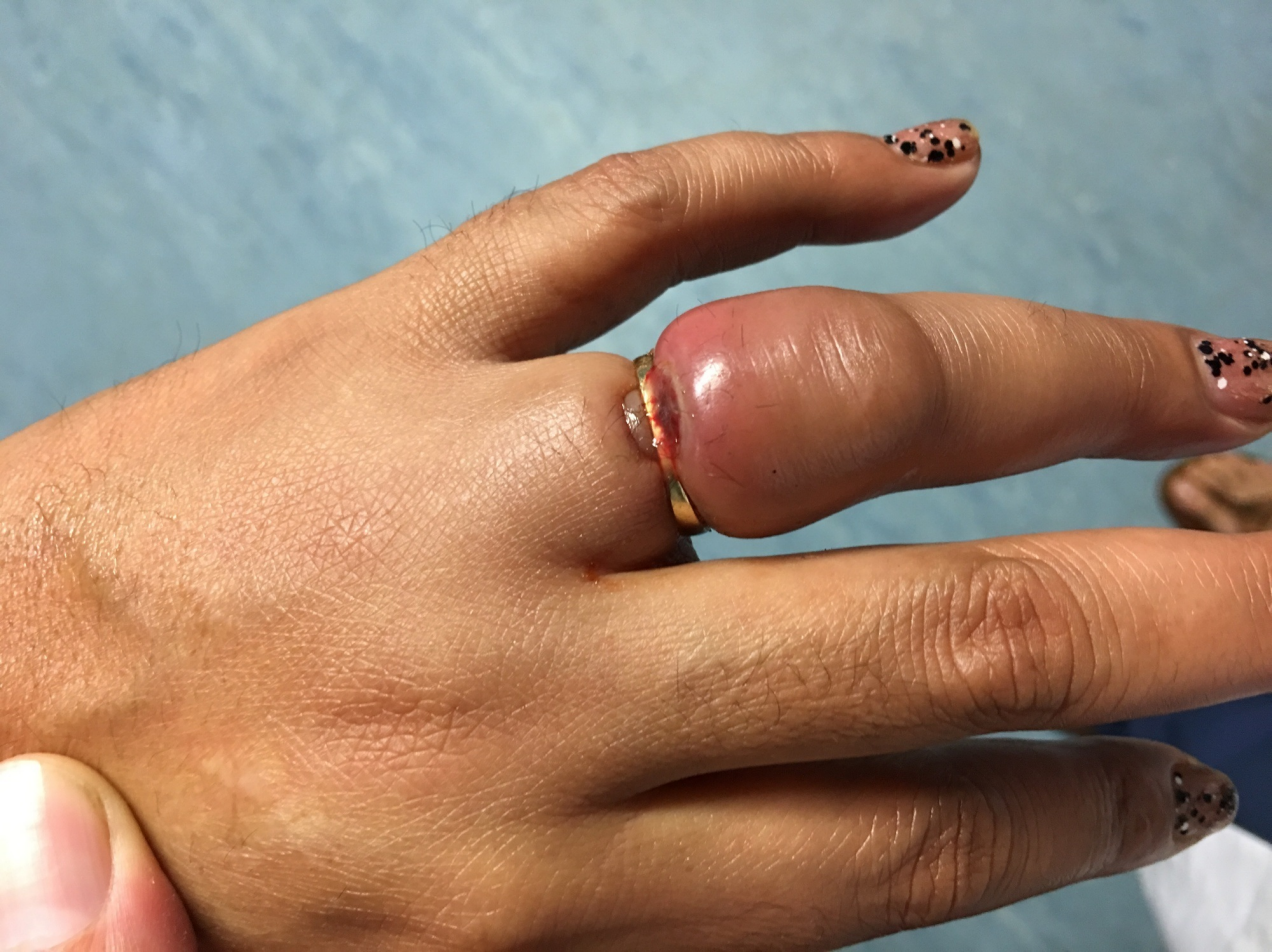
Swollen and painful left ring finger on presentation and prior to ring removal

**Figure 2:**
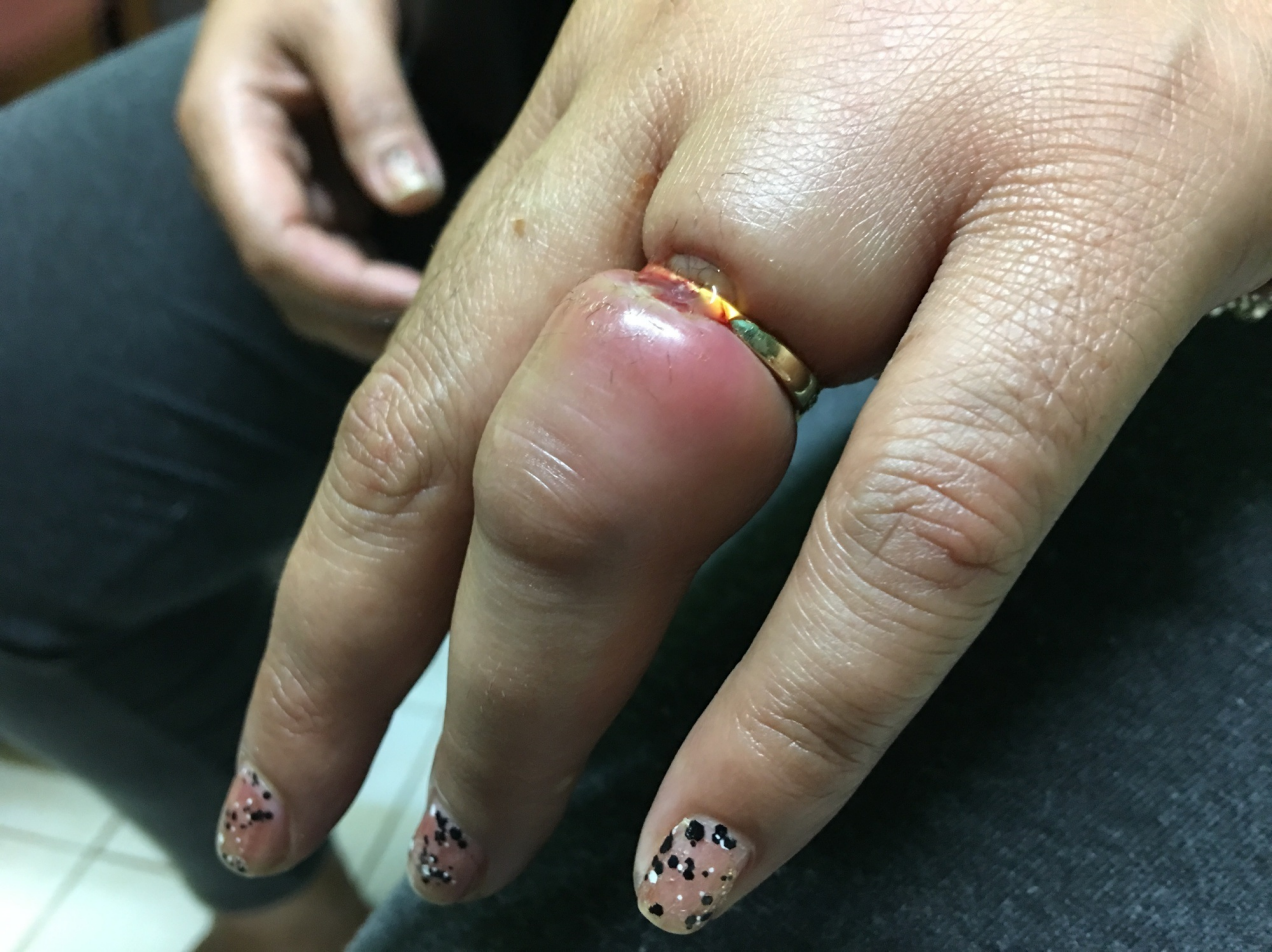
Swollen and painful left ring finger on presentation and prior to ring removal

The patient presented to the health outpost with aching of her left ring finger (pain score 8 out of 10) and associated swelling localized to the proximal phalanx (PIP) (Figures 1-2).

The patient first noticed the swelling in her ring finger three days prior, after a 10-hour flight. Her vitals were within normal limits. On examination of the finger, there was significant edema and shallow ulceration with granulation tissue formation over the ring margin. There was no purulent drainage, fluctuance, or streaking redness. Distal sensation was intact with normal two-point discrimination. The finger was warm, with capillary refill time less than two seconds. She was unable to flex the finger distal to the metacarpophalangeal joint.

**Figure 3:**
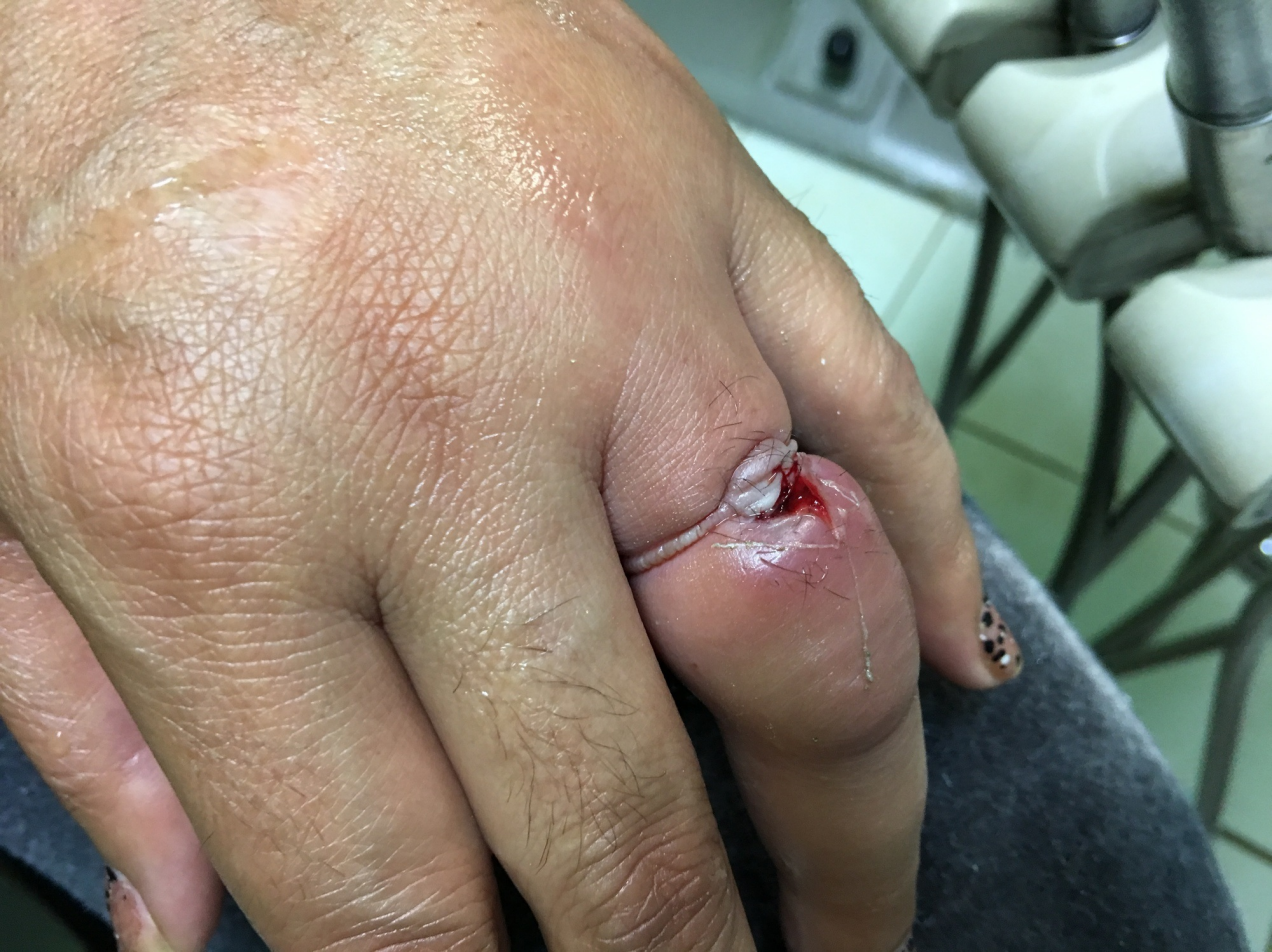
Left ring finger following ring tourniquet resolution and wound irrigation

Elevation of the limb, ice pack application, and lubrication with traction were all attempted. String technique was attempted but was not tolerated by the patient. The available topical anesthetic was insufficient to control the pain associated with string compression. Ring or bolt cutters were unavailable. The patient consented to ring cutting as ring preservation was not a priority. A topical anesthetic gel was applied to the finger. Successful ring removal was accomplished with a high-speed dental handpiece as well as water and a steel spatula to protect the underlying tissue (Figure 3) (Video 1).

**Video 1:** This video shows the use of a high-speed dental handpiece to resolve a ring tourniquet; several burrs were used to remove this gold and metal alloy wedding ring from the patient’s finger

A course of oral antibiotics was prescribed and tetanus toxoid vaccination was prophylactically administered following the ring removal [1-3]. On post procedure examination, the digit had intact distal sensation, capillary refill time less than two seconds, and improved range of motion. The patient had a full recovery without any complications or loss of function upon follow-up at four weeks.

## Discussion

In this case, we demonstrated an appropriate situation and technique for the resolution of a ring tourniquet with a high-speed dental tool. While this technique has been previously described as a tertiary option in a well-stocked office or emergency department, in resource-limited settings, it is reasonable to utilize a high-speed dental tool or another rotary tool with an appropriate cutting after the failure of ring sparing techniques. The rapid resolution of ring tourniquet prevents complications including nerve damage, necrosis, and gangrene following prolonged ischemia. The primary risk of this technique is the possibility of harm to the patient from the high-speed tool, either from direct trauma from the tool or thermal injury. However, as we have demonstrated, protection with a steel spatula and water to cool the instrument can help mitigate some of this risk. The early use of a high-speed dental drill or rotary tool for the treatment of ring tourniquet syndrome serves patients well after failure of ring sparing techniques.

## Conclusions

For patients presenting with a ring tourniquet syndrome, the early use of an immediate and precise cutting tool with the appropriate technique may optimize patient outcomes and allow for rapid resolution.
